# Implementing a Stratified Vocational Advice Intervention for People on Sick Leave with Musculoskeletal Disorders: A Multimethod Process Evaluation

**DOI:** 10.1007/s10926-021-10007-6

**Published:** 2021-10-04

**Authors:** Fiona Aanesen, Britt Elin Øiestad, Margreth Grotle, Ida Løchting, Rune Solli, Gail Sowden, Gwenllian Wynne-Jones, Kjersti Storheim, Hedda Eik

**Affiliations:** 1 Department of Physiotherapy, Oslo Metropolitan University, Oslo, Norway; 2 Research and Communication Unit for MSK Health (FORMI), Division of Clinical Neuroscience, Oslo University Hospital, Oslo, Norway; 3 School of Medicine, Faculty of Medicine and Health Sciences, Keele University, Keele, UK; 4 Connect Health, Newcastle upon Tyne, UK; 5 School of Medicine, and School of Nursing and Midwifery, Faculty of Medicine and Health Sciences, Keele University, Keele, UK

**Keywords:** Vocational rehabilitation, Musculoskeletal diseases, Sick leave, Return to work, Process evaluation

## Abstract

*Purpose* To perform a process evaluation of a stratified vocational advice intervention (SVAI), delivered by physiotherapists in primary care, for people on sick leave with musculoskeletal disorders participating in a randomised controlled trial. The research questions concerned how the SVAI was delivered, the content of the SVAI and the physiotherapists’ experiences from delivering the SVAI. *Methods* We used qualitative and quantitative data from 148 intervention logs documenting the follow-up provided to each participant, recordings of 18 intervention sessions and minutes from 20 meetings with the physiotherapists. The log data were analysed with descriptive statistics. A qualitative content analysis was performed of the recordings, and we identified facilitators and barriers for implementation from the minutes. *Results* Of 170 participants randomised to the SVAI 152 (89%) received the intervention and 148 logs were completed. According to the logs, 131 participants received the correct number of sessions (all by telephone) and 146 action plans were developed. The physiotherapists did not attend any workplace meetings but contacted stakeholders in 37 cases. The main themes from the recorded sessions were: ‘symptom burden’, ‘managing symptoms’, ‘relations with the workplace’ and ‘fear of not being able to manage work’. The physiotherapists felt they were able to build rapport with most participants. However, case management was hindered by the restricted number of sessions permitted according to the protocol. *Conclusion* Overall, the SVAI was delivered in accordance with the protocol and is therefore likely to be implementable in primary care if it is effective in reducing sick leave.

## Introduction

Musculoskeletal disorders include injuries and disorders affecting joints, bones and soft tissues [1] and are major contributors to years lived with disability worldwide [2]. In Norway, musculoskeletal disorders are the main cause of sick leave and are associated with a significant burden on individuals and economic costs to society [3]. Sick leave is influenced by several factors such as individual health and coping strategies, healthcare provision, social security systems and workplace factors [4–8], and vocational interventions should aim to identify and overcome individual obstacles to return to work (RTW) [8, 9]. Cullen and colleagues [10] reviewed intervention and cohort studies on the effectiveness of workplace interventions on RTW and recommended multi-domain interventions including work modification, health care provision and service coordination. In a meta-ethnography, Grant and colleagues [8] identified common barriers to RTW for people with chronic pain. They proposed that RTW interventions should be individualised and focus on collaboration with the person on sick leave and their employer, to find ways to manage pain at the workplace. Moreover, they suggested that interventions could be delivered by case managers located in primary health care [8].

An individually tailored RTW intervention delivered by case managers in primary care was effective in reducing work absence, compared to best current care for people with musculoskeletal pain in the UK [the Study of Work And Pain (SWAP) trial] [11, 12]. The intervention included advice about health and work, service coordination and stepped care. However, the intervention has not been tested in countries with other health and welfare systems. Therefore, we developed a stratified vocational advice intervention (SVAI), suitable for Norway, based on the SWAP intervention. The SVAI was delivered by physiotherapists in primary care, to people on sick leave with musculoskeletal disorders participating in a randomised controlled trial (RCT) in Norway (the MI-NAV study) [13]. The SVAI meets the Medical Research Councils (MRC) criteria for complex interventions as it is individually tailored and potentially involves cooperation with several stakeholders [14]. The MRC recommend performing process evaluations of complex interventions [14] to provide information about the intervention delivery and contextual factors that may influence the study results [14–16]. Integrating process and outcome data can provide insights into why an intervention is successful or why it fails to work and whether it is feasible to implement the intervention in daily practice [17–19]. The overall aim of this study was to perform a process evaluation of the delivery of the SVAI in the MI-NAV study. Our research questions were:

1. How was the SVAI delivered?
  - What training and resources were provided to the physiotherapists who delivered the SVAI?
  - How many of the eligible study participants received the SVAI?
2. What was delivered in the SVAI?
  - What was discussed in the SVAI conversations?
  - Which elements of the SVAI were delivered?
  - Was the SVAI delivered in accordance with the protocol and logic model?
3. What were the physiotherapists’ experiences of delivering the SVAI?

## Methods

The process evaluation is a multimethod study using both qualitative and quantitative process data to answer the different research questions [20]. We followed the MRC guidance for process evaluations of complex interventions [15] including a description of: *adaptations* made to the intervention, *training* and *resources* provided, *reach* (how many in the target group received the intervention), *dose* (how much of the different elements of the intervention was delivered) and *fidelity* (the extent to which the intervention was delivered according to the protocol) [15]. The results of the study are reported in accordance with the reporting criteria for the development and evaluation of complex interventions in health care (CReDECI 2) [21].

### The MI-NAV Study

**Fig. 1:**
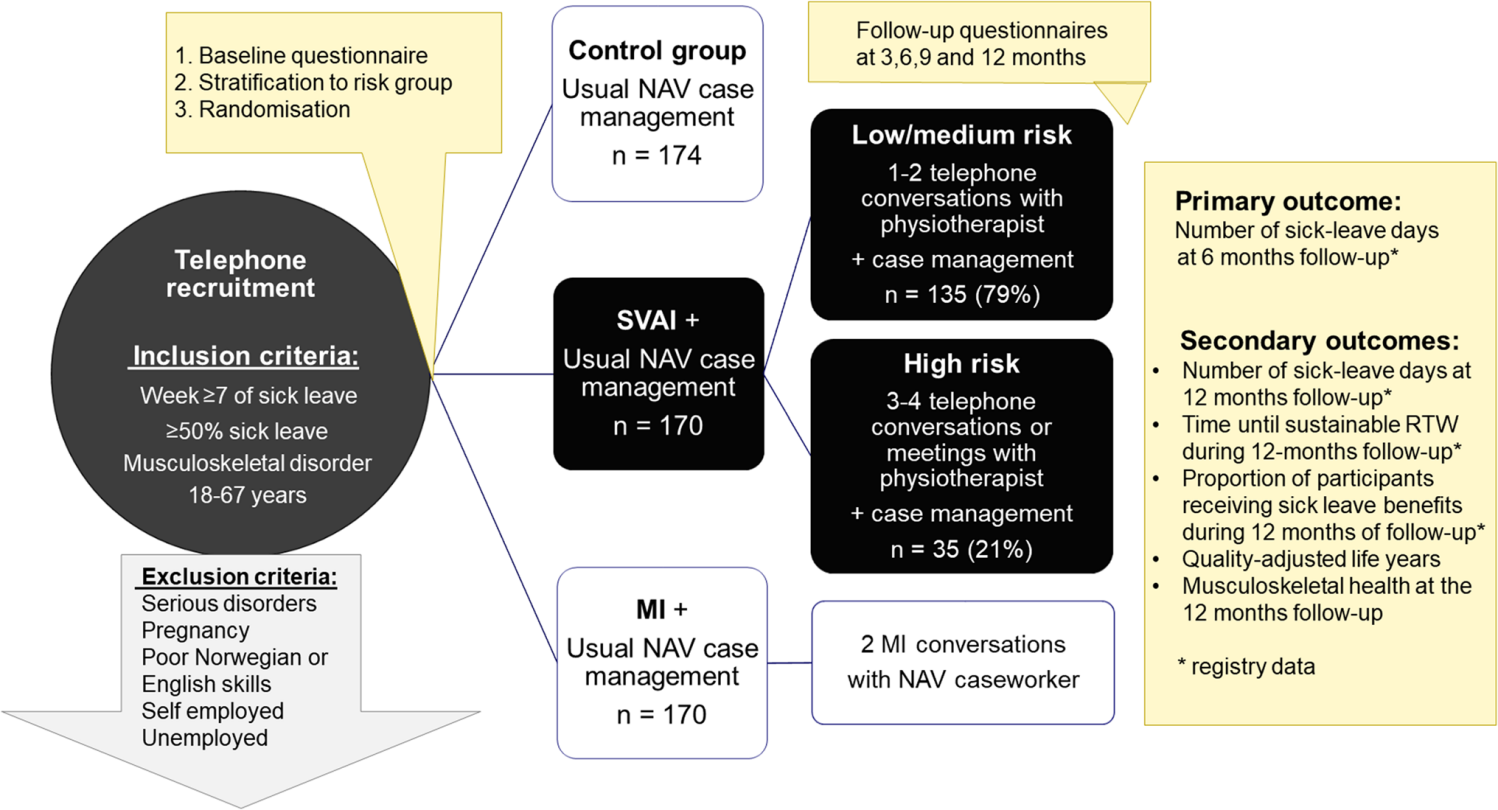
Illustration of the MI-NAV study. The black boxes describe the stratified vocational advice intervention (SVAI). *NAV* Norwegian Labour and Welfare Administration, *MI* motivational interviewing, *RTW* return to work

The MI-NAV study included a randomised controlled trial (RCT) with three arms in which all participants received usual follow-up from the Norwegian Labour and Welfare Administration (NAV). In addition, participants in the intervention arms received either motivational interviewing (MI) delivered by NAV caseworkers or the SVAI delivered by physiotherapists. The RCT was conducted in the South-East of Norway and has been described in detail in the study protocol [13] and at ClinicalTrials.gov (identifier: NCT03871712). Figure 1 shows an overview of the trial with the inclusion and exclusion criteria. The results of the outcome assessments, economic evaluations and mediation analyses of the SVAI and MI will be reported later. The Regional Committee for Medical and Health Research Ethics reviewed the study protocol and concluded that the study did not require approval, as it does not generate new health research (2018/1326/REK sør-øst A). The study was approved by the Norwegian Centre for Research Data (identifier: 861249) and conducted according to the Helsinki declaration and the General Data Protection Regulation. Participation was voluntary and did not influence sick leave benefits. Written informed consent was obtained from all participants prior to inclusion, and an additional consent was obtained to make recordings of the intervention sessions.

### Interventions

#### Usual Follow-Up

In Norway, employees with certified sick leave are entitled to full wage replacement for up to 1 year. The first 16 days are covered by the employer, the rest by the National Insurance Scheme administered by the NAV [22]. According to the NAV’s guidelines, the employer and employee have the main responsibility for the sick leave follow-up and should meet and make a follow-up plan within 4 weeks of the start of sick leave [22, 23]. Also, the employer is responsible for arranging a dialogue meeting with the employee within 7 weeks of the start of sick leave [23]. Within 26 weeks of the start of sick leave, the local NAV office is responsible for organizing a second dialogue meeting with the employee, the employer and the sick-leave certifier (when necessary) [23]. The NAV can also arrange a third dialogue meeting to assess the need for work-related measures within one year of sick leave [23].

#### Stratified Vocational Advice Intervention (SVAI)

The SVAI is an adaptation of the vocational advice intervention developed for the SWAP trial [12]. The intervention emphasizes the identification and problem solving of modifiable health and work-related obstacles to RTW [12]. The main adaption made to the intervention in the MI-NAV study was that the participants were stratified into two risk groups before random allocation (low/medium or high-risk for long-term sick leave) [13] and follow-up was customised according to risk group. Whereas, the SWAP intervention was delivered as stepped care and follow-up was increased (stepped up) depending on the participant’s needs [11]. Recruitment and inclusion criteria also differed between the two trials. In the SWAP trial the participants were recruited through their general practitioner (GP) and could have shorter sickness absence or still be at work (but struggling) [11]. In the MI-NAV trial participants were on sick leave for ≥ 7 weeks and self-employed workers were not included, as the evaluation of the SWAP trial showed that the vocational advice was less helpful for this group [24]. Another reason for excluding self-employed workers was that they receive extra follow-up from the NAV [25]. Reasons for excluding participants on short time sick leave were that subgroup analyses from the SWAP trial showed that the intervention was most effective for participants with ≥ 10 days of sickness absence compared to those with shorter absence [11]. Also, more than 80% of all people on sick leave in Norway RTW before week eight of the sick leave period [26].

The SVAI was a low intensity intervention consisting of case management provided by trained physiotherapists. The physiotherapists received a detailed manual on how to deliver the SVAI, and were asked to follow a semi-structured conversation guide including 15 core questions to clarify the participants’ current health and work situation (Appendix 1). According to the MI-NAV study protocol, the low/medium risk group should be offered 1–2 phone calls (lasting up to one hour) to identify obstacles to RTW, provide evidence-based advice on the management of musculoskeletal pain (in the context of work), support problem solving to overcome modifiable obstacles to RTW, collaboratively agree goals for RTW and develop and implement an action plan. The high-risk group should be offered 3–4 sessions with the physiotherapist, the first by telephone and the remaining sessions either by phone or as face-to-face meetings, including an optional worksite meeting. The content of the SVAI sessions was the same for the two risk groups. In addition, the physiotherapists should facilitate communication, collaboration and coordination with stakeholders and signpost to other services if necessary. The duration of the follow-up period was flexible but should end by week 26 of the participants’ sick leave, as this is when the NAV becomes more involved in the sick leave follow-up. The treatment targets, intervention components and theoretical underpinnings of the SVAI are described in the SVAI logic model (Appendix 2).

### Training of the Physiotherapists Delivering the SVAI

**Fig. 2:**
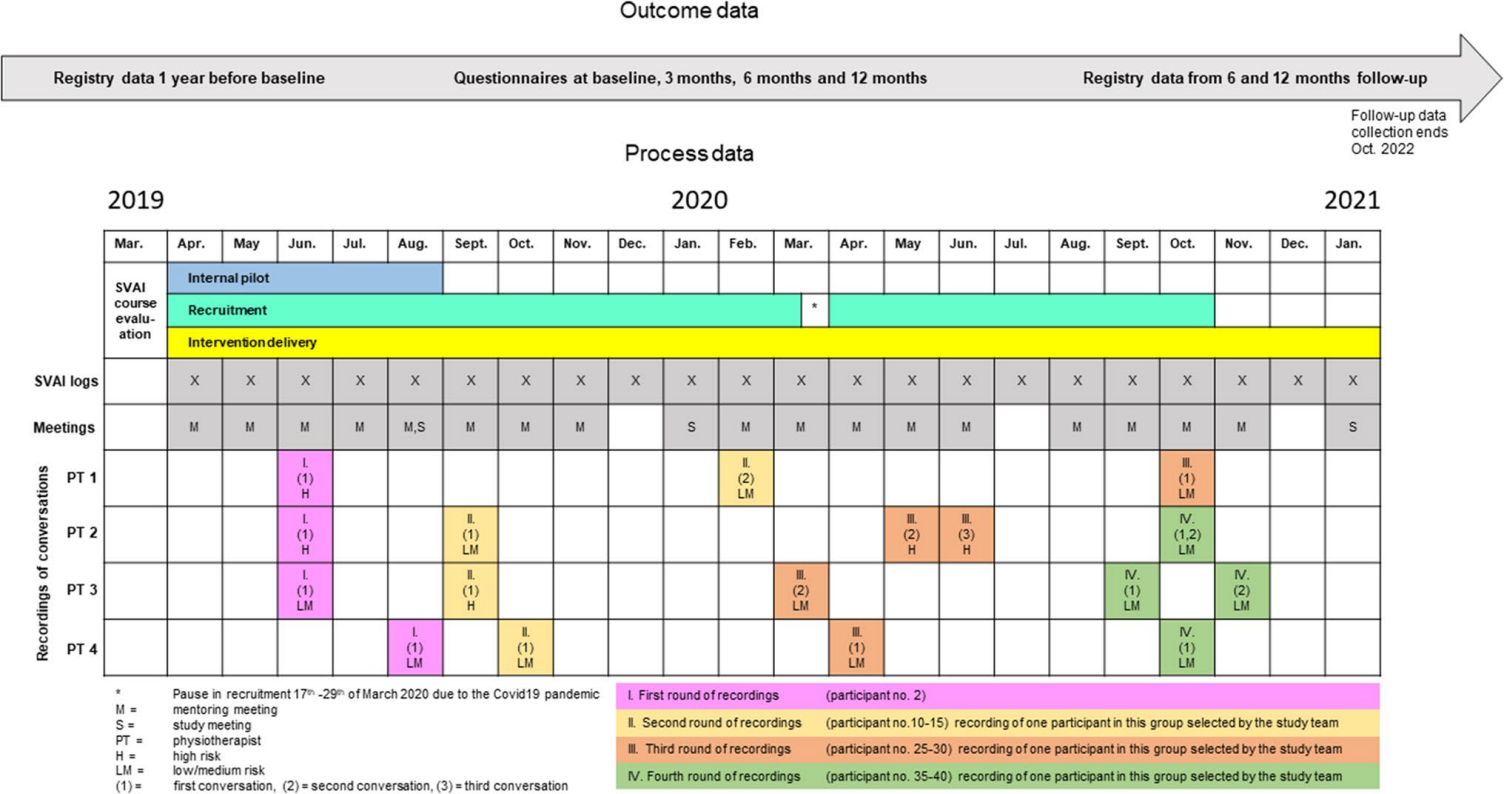
Timeline for recruitment and data collection in the MI-NAV Study

The training in the SVAI was a 3 + 2-day course led by one of the authors (GS). The course consisted of presentations, discussions and role-play covering topics such as: sick leave follow-up in Norway, the relationship between health and work, communication skills, identifying and addressing obstacles to RTW (through the provision of information and advice, problem solving, goal setting, case management and action planning). The study team held online mentoring sessions with the group of physiotherapists every month during the intervention period (except December and July, due to holidays). In addition, three meetings were held to discuss the study proceedings with the entire study group (including caseworkers and administrators from the NAV) (Fig. 2).

#### Resources

The physiotherapists were given a summary aide memoir of possible actions to support the participants to overcome common obstacles to RTW. They also had online sources of information about pain management, mental health, sleep, social work issues, sick leave benefits and follow-up from the NAV. In addition, they had three types of leaflets with information about the study and evidence-based information about work and health. The physiotherapists could distribute the leaflets to participants, employers and health care professionals if the participants consented.

#### Collection of Process Data

The data were collected before and during the intervention period of the MI-NAV study (Fig. 2). The physiotherapists filled out evaluation forms from the SVAI training and provided information about their work experience. To obtain information about the content of the SVAI sessions, audio recordings were made of telephone conversations between the 4 main intervention deliverers and 10% of the study participants who received the SVAI. The physiotherapists were asked to record conversations at regular intervals during the intervention period (Fig. 2), and to fill in information in an intervention log every time they had contact with a participant (one log per participant). The physiotherapists used the logs to document the participants’ responses to questions about their current work and health situation and obstacles to RTW. The logs also included information about number, length and types of contact with the participants, action plans and type of case management provided. Information concerning the physiotherapists’ experiences from delivering the SVAI was gathered from minutes from mentoring meetings and meetings with the entire study group.

#### Data Analysis

The qualitative analyses were performed by two of the authors (FA and HE). The recordings of the SVAI sessions were transcribed verbatim, and a descriptive content analysis of the conversations was performed, inspired by Braun and Clarke’s framework for thematic analysis [27], using the software QSR Nvivo 12. First, we listened to the recordings and read the transcripts to get familiar with the data, then the data were coded, and themes were developed from the coded data. The quantitative data from the SVAI logs were analysed with descriptive statistics including frequencies, percentages, means and median values using SPSS version 27. The data from the analyses were combined to describe fidelity to the SVAI, including an appraisal of whether the conversations covered the core topics in the conversation guide and whether the intervention elements described in the logic model and protocol were delivered by the physiotherapists. Additionally, we assessed if the time until the first contact, the number and length of the sessions and the development of RTW goals and action plans were performed in accordance with the protocol. The analysis of the mentoring and meeting minutes was guided by the analytical question: ‘What did the physiotherapists experience as facilitators and barriers when delivering the SVAI?’ All the analyses of the process data were performed prior to the outcome evaluation of the trial.

## Results

### Recruitment and Reach

Researchers employed by the NAV directorate contacted workers on sick leave by telephone. Eligible participants wanting to take part in the study received a link to study information and signed informed consent forms, before answering the baseline questionnaire. Participants scoring ≥ 9 on the Keele STarT MSK tool [28] and ≥ 60 on the Örebro MSK Pain Screening Questionnaire Short Form [29] were stratified to the high-risk group, and those with lower scores on one or both of the questionnaires were stratified to the low/medium risk group [13]. A total of 514 participants (25% of all eligible candidates) were included in the trial between April 2019 and October 2020. The first phase of the study was an internal pilot to test study practicalities. As only minor changes were made during the pilot, the pilot participants (n = 101) were included in the main trial. In total, 170 participants were randomised to the SVAI, 135 (79%) in the low/medium risk group and 35 (21%) in the high-risk group. Eighteen participants did not receive the SVAI: eight had RTW > 50% before the first phone call, five could not be reached, three were not contacted, one had been on sick leave for more than 26 weeks before the intervention commenced, and one withdrew from the study. The remaining 152 participants (89%) received the SVAI.

### Training and Background of the Physiotherapists

The course evaluations showed that all but one of the physiotherapists felt they had the skills to help participants identify and overcome obstacles to RTW, after finishing the SVAI training course. However, several of the physiotherapists would have liked more practice in conducting the SVAI conversations, especially the follow-up conversations. Eight physiotherapists completed the SVAI training (2 men, 6 women), but four withdrew early in the study due to other work commitments. The four remaining physiotherapists were all women aged between 28–45 years with 4–21 years of work experience in primary care. These four physiotherapists provided the SVAI to 95% of the participants (30–40 participants each).

### Recordings of the SVAI Sessions

#### Characteristics of the Study Participants in the Recordings

During the study, 18 recordings were made of conversations with 15 different participants, nine women and six men, mean age 48.6 years (range 35 to 63). Four were in the high-risk group and eleven in the low/medium risk group. Ten were blue-collar workers, three worked in the health sector and two had office jobs. They had a range of musculoskeletal conditions in different anatomical areas of the body. The sample was representative of the total SVAI cohort regarding age, sex and occupation, however 6% more were in the high-risk group.

#### Main Themes Discussed by the Participants in the Recorded SVAI Sessions

The participants’ descriptions of their health situation were related to two main themes, the first theme was *‘symptom burden’.* Pain was their main symptom and it affected their lives in many ways. They avoided certain activities and movements that aggravated their pain such as sitting, walking or lifting. For many the pain affected their sleep, was associated with fatigue and limited their ability to work and be social. The second theme was ‘*managing symptoms’*. The participants used different coping strategies such as using medication and different aids. Several emphasised the importance of finding a balance between activity and rest, and that the sick leave gave them the opportunity to exercise and time to get treatment. Many were searching for a diagnosis and had spent a long time waiting for health examinations and treatments. They described a feeling of standing still and that improvement was slow.

There were also two main themes related to RTW. The first was ‘*relations with the workplace’*. Most of the participants were satisfied with their work situation and wanted to return to their pre-sick leave hours of work and workplace. The amount of contact they had with the workplace varied. Some reported having regular, supportive contact with their employer and an effective follow-up plan in place. Others had a plan that was not being implemented, and some had received little support from their workplace and had no follow-up plan. The options for modified work (e.g. hours, roles, responsibilities, tasks) varied. Some had received support to commence modified work whilst others found it difficult to modify, either because of the nature of their work or because they perceived their employers as being unwilling to help. The second theme related to RTW was *‘fear of not being able to manage work’*. The main obstacle to RTW described by the participants was that they were afraid they would not be able to manage to do their work and that their symptoms or health problems would increase if they RTW too soon. Some felt they would not manage to RTW because of the intensity of their pain and fatigue. They found it difficult to combine working with engaging in exercise and treatment because they spent all their energy at work. Some had been told by health care professionals to take time to recover before they RTW and several wanted reassurance that it was safe to RTW with their health problems.

### Information from the Intervention Logs

The physiotherapists completed logs for 148 (97%) of the participants who received the SVAI, of these 114 (77%) were in the low/medium risk group and 34 (23%) in the high-risk group. The data from the SVAI logs are presented in Tables 1 and 2. All the follow-ups were provided over the telephone, the mean number of conversations was 2.0 (SD 0.5) in the low/medium risk group and 3.1 (SD 0.9) in the high-risk group. In total, the physiotherapists had documented contact with other stakeholders in 25% of the logs, 23 (20%) in the low/medium risk group and 14 (41%) in the high-risk group. The contacts were primarily referrals to treating physiotherapists or professionals working in “healthy life centres” (providing help with lifestyle changes).

**Table 1:** Description of the intervention elements delivered by the physiotherapists

Variable	All participants (%)	Low/medium risk group (%)	High risk group (%)
n (%)	148 (100)	114 (77)	34 (23)
Number of phone sessions			
1	13 (9)	12 (11)	1 (3)
2	106 (71)	96 (84)	10 (29)
3	10 (7)	1 (1)	9 (27)
4	19 (13)	5 (4)	14 (41)
Action plans	146 (99)	112 (98)	34 (100)
Information leaflets distributed			
To participant	8 (5)	3 (3)	5 (15)
To employer	7 (5)	3 (3)	4 (12)
To health care professionals	3 (2)	2 (2)	1 (3)
Contact with stakeholders ^a^	37 (25)	23 (20)	14 (41)
Employer	4 (3)	1 (1)	3 (9)
NAV	4 (3)	1 (1)	3 (9)
General practitioner	2 (1)	1 (1)	1 (3)
Physiotherapist	25 (17)	15 (13)	10 (29)
Other health care professionals ^b^	12 (8)	10 (9)	2 (6)
Several stakeholders ^c^	10 (7)	5 (4)	5 (15)

The data presented in the table are from the SVAI logs

*NAV* Norwegian Labour and Welfare Administration

^a^Any type of contact including arranging an appointment for the participant

^b^Mainly professionals from Healthy Life Centres, providing help with lifestyle changes

^c^Cooperated with two different stakeholders

**Table 2:** Timing and duration of the SVAI follow-up and number of core questions with information

Variable	n	All participants		Low/medium risk group		High risk group	
		Mean (SD)	Median (min–max)	Mean (SD)	Median (min–max)	Mean (SD)	Median (min–max)
Days until first contact	120	**2.2** (2.9)	**1** (0–13)	**2.3** (3.1)	**1** (0–13)	**1.7** (2.3)	**0** (0–7)
Days until first session	124	**5.8** (4.5)	**5** (0–36)	**6.0** (4.8)	**5** (0–36)	**5.2** (3.1)	**5** (1–13)
Intervention period (*days*)	123	**50.0** (27.0)	**42** (4–128)	**42.4** (21.0)	**39** (4–108)	**73.8** (29.9)	**74** (20–128)
Duration of sessions (*min*.)							
First	145	**47.1** (15.4)	**45** (20–90)	**45.6** (14.1)	**45** (20–90)	**52.2** (18.5)	**45** (30–90)
Second	116	**26.9** (12.6)	**25** (5–75)	**26.3** (12.5)	**25** (5–75)	**28.7** (12.9)	**30** (5–60)
Third	24	**29.2** (13.2)	**30** (15–75)	**35.0** (22.9)	**30** (20–75)	**27.6** (9.6)	**30** (15–45)
Fourth	12	**26.3** (12.8)	**25** (10–45)	**30.0** (18.0)	**35** (10–45)	**25.0** (11.7)	**20** (15–45)
Information on core questions ^a^	148	**14.1** (1.0)	**14** (9–15)	**14.1** (1.0)	**14** (9–15)	**14.3** (0.8)	**15** (12–15)

Mean and median values are given in bold

Mean and median values are included in the table as the variables were not normally distributed

The data presented in the table are from the SVAI logs

*(min-max)* (minimum-maximum), *min.* minutes

^a^Information noted against the core questions from the conversation guide (maximum 15)

### Fidelity to the Protocol

The protocol stipulated that the physiotherapists should contact participants within 7 days after randomisation, this occurred in 94% of cases, and 95% of the conversations lasted 60 min or less, in keeping with the protocol. In total, 89% of the participants received the correct number of conversations. However, 32% in the high-risk group received less than three conversations and 5% in the low/medium risk group received more than two conversations. The main reason for this was that 18 participants were stratified to the wrong risk group by error. Seven with high-risk were wrongly classified to the low/medium risk group and eleven with low/medium risk were wrongly classified to the high-risk group.

All the SVAI logs had documented information against ≥ 9 of the 15 core questions in the conversation guide (mean 14.1, SD 1.0) (Table 2). The information most often missing from the logs (41% missing) was the participants’ contact with the NAV. Data from the content analysis of the recorded sessions, showed that the physiotherapists predominantly provided information and reassurance regarding self-management of symptoms and musculoskeletal ill health and tried to reduce the participants’ fear avoidance behaviours. This included information about body structures, normal age-related changes and factors that could affect the pain experience. The physiotherapists emphasised the importance of physical activity and suggested a gradual increase in activity. Additionally, they advised several of the participants to seek physiotherapy treatment or to contact their GP. In some cases, they stepped out of their role as vocational advisers and provided advice to participants as clinical physiotherapists. Concerning RTW, they advised the participants to stay in contact with their workplace and to make a follow-up plan with their employer or to revise the plan if needed. They also gave the participants information about their rights in terms of requesting dialogue meetings with their employer and the NAV. However, the physiotherapists did not attend any workplace meetings and rarely liaised with the participants’ employer, GP or the NAV (Table 1). The recordings showed that the physiotherapists suggested a gradual RTW to many of the participants, primarily involving starting with fewer hours of work and building this up over time. If the participants were struggling with certain tasks, they recommended that they discuss this with their employers and explore options for modified work. They also gave reassurance that it was safe to RTW and that it was normal for symptoms to temporarily increase as they RTW or increased their workload. The physiotherapists discussed RTW goals with the participants and made action plans. This was confirmed in the SVAI logs where 93% of the logs included descriptions of work goals (short-term goals, long-term goals or both). Only two logs did not include an action plan (Table 1), and 94% of the action plans included notes to show that the plan had been reviewed.

### Experiences from Delivering the SVAI

Twenty meetings were held with the physiotherapists where they discussed cases and experiences from delivering the SVAI (Fig. 2). Overall, the meetings had high attendance from the four main intervention deliverers. Table 3 gives an overview of the facilitators and barriers for implementation discussed during the mentoring. The main facilitator described by the physiotherapists was the mentoring, while the main barrier was being restricted to providing two telephone sessions for the low/medium risk group. Additionally, the lack of meeting facilities made it difficult to arrange face-to-face meetings. As half of the physiotherapists withdrew from the study, the remaining four had to cover a large geographical area and did not have meeting facilities close to the participants. We made some changes during the pilot study in response to the physiotherapists’ feedback. For example, simplifying the conversation guide and taking action to increase recruitment to the trial. To increase the focus on RTW, the order of the questions in the guide was changed so that questions regarding work came first. A NAV caseworker participated in one of the mentoring sessions to answer questions regarding benefits from the NAV.

**Table 3:** The physiotherapists experiences from delivering the SVAI

Facilitators/positive experiences	Barriers/challenges
• The phone-conversations went well and it was easy to build rapport with most participants over the phone (5, 6, 20) • The help, advice and support provided in the SVAI appeared to be appreciated by the participants (4, 5, 7, 11, 12, 13, 20) • The physiotherapists perceived it as an advantage that they were independent from the NAV (7, 19) • Having been training as physiotherapists was an asset when giving the participants advice and reassurance about musculoskeletal symptoms (19) • The questions in the conversation guide gave the participants the opportunity to describe many aspects of their situation (6) • The support, advice and information provided during the mentoring sessions was helpful (3, 5, 9, 10) • A shared digital forum (facebook group) made it easy for the physiotherapists to cooperate and share tips between mentoring (7) • The physiotherapists appreciated receiving feedback on the sessions they recorded and learnt from listening to their own recordings of sessions with participants (13, 14)	• Slow recruitment of participants at some points in the study (1, 3, 11, 13, 14, 15) • Challenges in becoming familiar with the conversation guide because it included several overlapping questions (1, 6) • It was difficult to build rapport over the phone with people who were not motivated to RTW and with participants who did not have Norwegian as their first language (3, 11, 12) • There were some problems getting hold of participants (12) • The lack of meeting locations and long distances that participants would have had to travel to meeting locations was a barrier to arrange face-to-face meetings (1, 16) • Participants did not want workplace meetings or did not want the physiotherapists to attend workplace meetings (10, 12, 16, 20) • The physiotherapists did not feel comfortable contacting the participants employers because they did not feel they knew their situation well enough to discuss the work related issues with employers (19, 20) • The limit on the numbers of phone calls allowed made it difficult to help some participants in the low/medium risk group (3, 6, 16, 19) • It was challenging to understand what RTW support the NAV might have been able to provide and often the participants did not fit the criteria for the NAV’s schemes (9, 10, 11, 18) • It was hard to determine what health care to recommend to participants (2, 3, 13, 20) • It was difficult to encourage RTW or increased activity when the participant had received advice from other health care professionals to be careful/stay on sick leave (7, 12, 15, 20) • The physiotherapists did not feel comfortable questioning the treatment provided by other health care professionals (6, 10) • It was not possible to send information to participants by email or text message due to The General Data Protection Regulation (6, 10) • There were several barriers related to the Covid19 pandemic: less access to health care, many workplaces were closed, jobs were at risk and participants in the risk groups for getting seriously ill from Covid19 were afraid to get infected if they RTW (12, 13, 14, 15) • In a few cases the physiotherapists felt the participants were in the wrong risk group (2, 17, 20)

NAV = Norwegian Labour and Welfare Administration, RTW = return to work

The data presented in the table are from the meeting minutes. The numbers refer to the meetings were the topic was discussed. The meetings are numbered in chronological order (1 = first meeting etc.)

## Discussion

The physiotherapists received 5 days of training before delivering the SVAI and attended monthly mentoring meetings during the intervention phase of the RCT. Of the 170 participants randomised to the SVAI, 89% received the intervention. All the sessions were by telephone and covered the main topics in the conversation guide. The SVAI was mainly delivered in accordance with the protocol. However, the physiotherapist experienced that the restricted number of sessions permitted for the low/medium risk group hindered case management.

Despite an overall good fidelity to the SVAI, there were some of the intervention elements that were not delivered. Firstly, no face-to-face meetings were held. This was due to the lack of suitable meeting facilities and social distancing protocols implemented on the 13th of March 2020 by the Norwegian government, following the Covid-19 pandemic. Furthermore, most of the participants were in the low/medium risk group and therefore should not have face-to-face meetings. Although the physiotherapists felt they were able to build rapport with most participants over the telephone, they would have preferred to have face-to-face conversations with participants for whom Norwegian was a second language, as they found it more challenging to communicate with these participants. They also thought that having a face-to-face meeting would have made it easier to establish a good rapport with participants who were not motivated to RTW. As nonverbal communication is restricted during telephone conversations the lack of face-to-face meetings could reduce the quality of the communication, and might compromise the effectiveness of the SVAI for some participants. However, several studies have shown that patient satisfaction with remote management is high across a broad range of interventions [30], and that telephone follow-up is equivalent to face-to-face interventions for improving physical function and pain for people with musculoskeletal disorders [30, 31].

A second element that was poorly implemented in the SVAI was stakeholder collaboration. The physiotherapists had few contacts with important stakeholders such as GPs and employers and did not attend workplace meetings. In the SWAP trial the physiotherapists were located in GP practices and collaborated with the GPs [11, 24], however they had few contacts with employers and only attended one workplace meeting [11]. Communication between RTW stakeholders can be challenging [4, 32–34], and many of the barriers described by the SVAI physiotherapists are commonly experienced in vocational rehabilitation [32, 35]. Information from the mentoring minutes showed that the SVAI physiotherapists did not have confidence to contact employers, because they did not feel that they were in a position to discuss workplace modifications. In addition, several of the physiotherapists reported that participants did not want them to attend workplace meetings. The lack of communication with the employers may have reduced the potential effectiveness of the SVAI, as workplace factors can influence sick leave and RTW [4, 8]. Although several systematic reviews have underscored the importance of including the workplace in RTW interventions [7, 8, 10, 36, 37], two Norwegian studies did not find any added benefit on RTW of workplace meetings [38] or telephone conversations with employers [39]. One explanation for the lack of benefit could be that Norwegian employers and employees on fulltime sick leave are required to cooperate and make a follow-up plan [23]. Nevertheless, several of the participants in the MI-NAV Study had not had meetings with their employer, demonstrating that the guidelines and policies are not always followed. This is in line with findings from a recent study involving NAV caseworkers who experienced that employers rarely used the follow-up plans [33]. Furthermore, NAV caseworkers [33] and clinicians working in occupational rehabilitation clinics in Norway [40] have underscored the importance of liaising with GPs, employers and other stakeholders during the RTW process.

Although liaison with employers is important to facilitate RTW, many of the SVAI participants were unsure how to manage their musculoskeletal disorders. The main barrier to RTW described by the participants was fear that RTW would aggravate their symptoms, which is in line with findings from previous studies [8]. This highlights the need for evidence based input from health care professionals about the health benefits of good work [41, 42], and advice regarding fitness for work [42]. The SVAI physiotherapists felt that their clinical background was an asset when providing advice about the management of musculoskeletal disorders and reassurance that RTW was not harmful. Although it may be helpful for RTW coordinators to have knowledge about health conditions, they should address work issues rather than medical issues [43, 44]. Interviews with the physiotherapists in the SWAP trial showed that they gave advice on the management of musculoskeletal pain when they felt unsure about how to help resolve work difficulties [24]. This was also the case for the physiotherapists providing the SVAI. Therefore, having a background as a physiotherapist may be both an asset and a challenge when the role is to support people with musculoskeletal disorders to RTW. Furthermore, studies investigating competencies of RTW coordinators show that it is important to have knowledge of the legal rights and responsibilities of workers, workplace policies and insurance systems related to sick leave and RTW [43–45]. However, the physiotherapists delivering the SVAI found it difficult to get an overview of the RTW support available through the NAV. Therefore, it could be beneficial if the physiotherapists had a mentor working in the national insurance system to help with questions regarding work related laws, regulations and RTW schemes and benefits. Nevertheless, it is important that the physiotherapists are independent from the NAV, as workers on sick leave may be reluctant to disclose information that might affect decisions regarding sick leave benefits, if they believe clinicians are working for the national insurance system [40].

### Strengths and Limitations

This multimethod process evaluation was performed in accordance with the MRC guidelines. By combining several qualitative and quantitative data sources we could describe different aspects of the delivery of the SVAI in the RCT. The qualitative data from the recordings provided detailed insight into the content that was delivered in the SVAI sessions, and the quantitative data from the logs provided information about the dose and type of follow-up provided to almost all the participants receiving the SVAI. Another strength of the study is that the qualitative data were analysed by two researchers, and that all the analyses were performed before the outcome evaluation of the trial and have therefore not been influenced by the results of the RCT.

Although we had recordings of conversations with 10% of the participants receiving the SVAI, it would have been preferable to have recorded all the conversations and then drawn a random sample for analysis. However, this was not possible as not all the participants consented to being recorded, it would also have increased the burden on the physiotherapists. Another limitation is that we did not conduct interviews with the study participants due to limited resources. Therefore, we lack information about the acceptability or helpfulness of the SVAI to participants. However, this process evaluation builds on the data from the evaluation of the SWAP trial where researchers conducted interviews with study participants as well as with the vocational advisers and GPs involved in the study [24].

## Conclusions

The results of this process evaluation show an overall good fidelity to the SVAI and the sessions included most of the elements from the SVAI logic model. However, some elements of the intervention were not implemented including face-to-face meetings and meetings at the workplace. The physiotherapists providing the SVAI rarely contacted employers, GPs or the NAV. The process evaluation suggests that it would be feasible to implement the SVAI in primary care if it is effective in helping people with musculoskeletal disorder to RTW. To improve future implementation, one should consider increasing the number of sessions allowed between the physiotherapists and participants with low to medium risk of long-term sick leave, or to deliver the intervention as stepped care. It would also be important to ensure conveniently located meeting facilities.

## Data Availability

To avoid breaching participant confidentiality the datasets generated and analysed during the current study will not be made publicly available.

## Appendix 1: Core Questions from the SVAI Conversation Guide

**Table:**

Topics	Core questions
Work situation	Start of current sick leave (date)
	Percent of sick leave (at first consultation)
	Can you describe your current work situation?
Identify and address RTW obstacles	How are your symptoms affecting your ability to work?
	What are your main concerns about RTW?
	Have you had a dialogue meeting with your employer?
	What contact have you had with the NAV?
	Has your employer made a RTW plan?
	How happy are you with your work and workplace?
	What could be done at the workplace to help you RTW or increase your work hours?
Goal setting	Short term work goal
	Long term work goal
Health situation	Could you please tell me briefly about the main health problem that you are struggling with at the moment?
	How is your health condition affecting your day to day?
	Can you describe any treatment you are receiving or have received for your condition?

## Appendix 2: Logic Model: Stratified Vocational Advice Intervention (SVAI)

**Table:**

Treatment targets	Elements of the SVAI intervention	Theoretical underpinning
**PERSONAL** **Health** - Not accessing timely and appropriate healthcare - Poor co-ordination, communication and co-operation between health and other stakeholders **Psychosocial** *Cognitions*: - Unhelpful beliefs about health and work - Low RTW self-efficacy *Emotions:* - Anxiety about RTW - Anger/frustrations with workplace *Behaviours:* - Low levels of physical activity and participation in everyday life - Sleep pattern incompatible with work **OCCUPATIONAL/ORGANISATIONAL** - Suboptimal amount and nature of contact with the sick-listed employee - Excessive stressors at work and/or suboptimal ability of employee to respond adaptively to stress - Poor communication between internal workplace stakeholders - Lack of work adjustments/transitional arrangements - Lack of or poorly devised RTW plan - Poor implementation of RTW plans	**ASSESSMENT** To clarify the current health and work situation and any obstacles to RTW **EXPLORE THE VALUE OF WORK** To increase RTW motivation **PROBLEM SOLVING** To identify and overcome modifiable obstacles to RTW **Case management:** to facilitate communication, collaboration and coordination with stakeholders (e.g. to liaise with GP/HCP to facilitate referrals, agree on RTW plans and goals, to encourage contact with the workplace, to facilitate work modifications (if needed) and to set up and conduct worksite meetings **Education**: facilitate an evidence based understanding of symptoms and ill health in the context of work, understand the RTW process and options and address unhelpful beliefs or knowledge gaps **Advice and reassurance:** increase confidence to RTW, improve sleep quality and quantity and restore work consistent awake/sleep pattern **Graded activity/exposure:** promote active self-management, reduce fear-avoidance behaviour, behavioural re-activation, graded RTW (hours/ tasks/responsibilities) **Workplace modification:** temporary or permanent **Signposting to other services:** obtain assistance with work related issues (e.g. bullying or harassment), or wider social issues (e.g. debt, wage replacement benefits, housing) or obtain help with changing job/employer **Goal setting:** to identify and agree RTW and other goals, commit to agreed goals, to monitor progress, and provide feedback and encouragement to increase motivation and adherence **RTW planning and implementation:** to develop a written action plan, provide support, monitor progress and problem solve difficulties	**SOCIAL COGNITIVE THEORY** - Beliefs about capabilities of RTW - Beliefs about health consequences of performing the behaviour - Skills: instructions on how to perform a behaviour: development, competence, ability, practice - Behavioural regulation: action planning and self-monitoring to change actions **SELF-DETERMINATION THEORY** - Support *intrinsic motivation* to RTW by exploring what the participant value about their work - *Relatedness:* clarify situation from the participants’ perspective. Facilitate communication with the workplace - *Competence* to RTW: provide education, advice, reassurance, problem solve and support participant to develop/use skills to overcome RTW barriers - *Autonomy support:* collaborate closely with participant to agree goals, plan RTW and empower the participant to take direct action **THE COMMON-SENSE MODEL OF SELF REGULATION** - Identify participants’ beliefs about their health problems, treatment and management strategies - Improve knowledge about health and work, reduce fear avoidance and promote active self-management

*RTW* return to work, *GP* general practitioner, *HCP* health care professionals.
